# Pulmonary echinococcosis mimicking multipl lung metastasis of breast cancer: The role of fluoro-deoxy-glucose positron emission tomography

**DOI:** 10.1186/1477-7819-6-7

**Published:** 2008-01-21

**Authors:** Yavuz Kurt, İlker Sücüllü, Ali İlker Filiz, Muammer Urhan, Mehmet Levhi Akın

**Affiliations:** 1 Department of General Surgery, GATA Haydarpasa Istanbul, Turkey; 2 Department of Nuclear Medicine, GATA Haydarapasa Istanbul, Turkey

## Abstract

**Background:**

Echinococcosis is still a serious problem particularly in endemic areas such as South and Central America, Mediterranean countries, and Russia. Furthermore, hydatid cysts of the lung are often indistinguishable from a variety of other pulmonary lesions such as lung tumors

**Case presentation:**

We herein present a 56 year old woman with breast cancer who presented with bilateral pulmonary nodules due to echinococcosis granulosis that mimicked metastatic breast cancer to the lung.

**Conclusion:**

During the evaluation of the malignancies which could metastasize to the lung, it must be kept in mind that the appearance of bilateral multiple pulmonary masses can also be the sign of a pulmonary echinococcosis especially in endemic areas. FDG-PET with its known high negative predictive value in characterizing indeterminate pulmonary nodules >1 cm is very helpful to characterize this kind of lesions.

## Background

Echinococcosis is a parasitic infestation caused by larvae of the tapeworm echinococcus. Although there are four species the vast majority of hydatid disease in humans are caused by echinococcus granulosis which causes cystic echinococcosis and has a worldwide distribution. Echinococcus multilocularis causes alveolar echinococcosis which can be seen less frequently in humans. In cystic echinococcosis humans are an aberrant host and are usually infected by oral ingestion of excrement from an infected dog. Infected red fox has an important role for alveolar echinococcosis [1,2]. Parasitic eggs turned to larvae form after ingestion in the intestine and migrate to the other organs by penetrating intestinal wall and then develop cystic or alveolar echinococcosis. The liver and lungs are the most common involved organs. Hydatid cysts may rupture, can become secondarily infected, or may infect other organs. Alveolar echinococcosis behaves like a malignant tumor biologically. Humans Echinococcosis is still a serious problem particularly in endemic areas such as South and Central America, Mediterranean countries, Australia and Russia. Furthermore, hydatid cysts of the lung are often indistinguishable from a variety of other pulmonary lesions such as lung tumors [2-4].

## Case presentation

**Figure 1:**
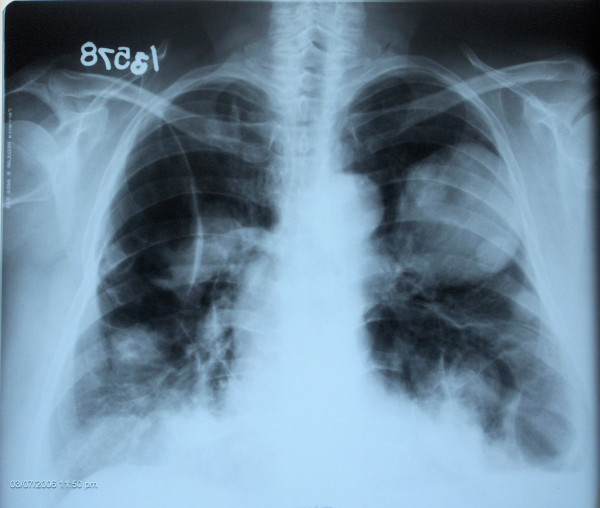
Chest radiography shows multiple nodules and masses involving both lungs.

**Figure 2:**
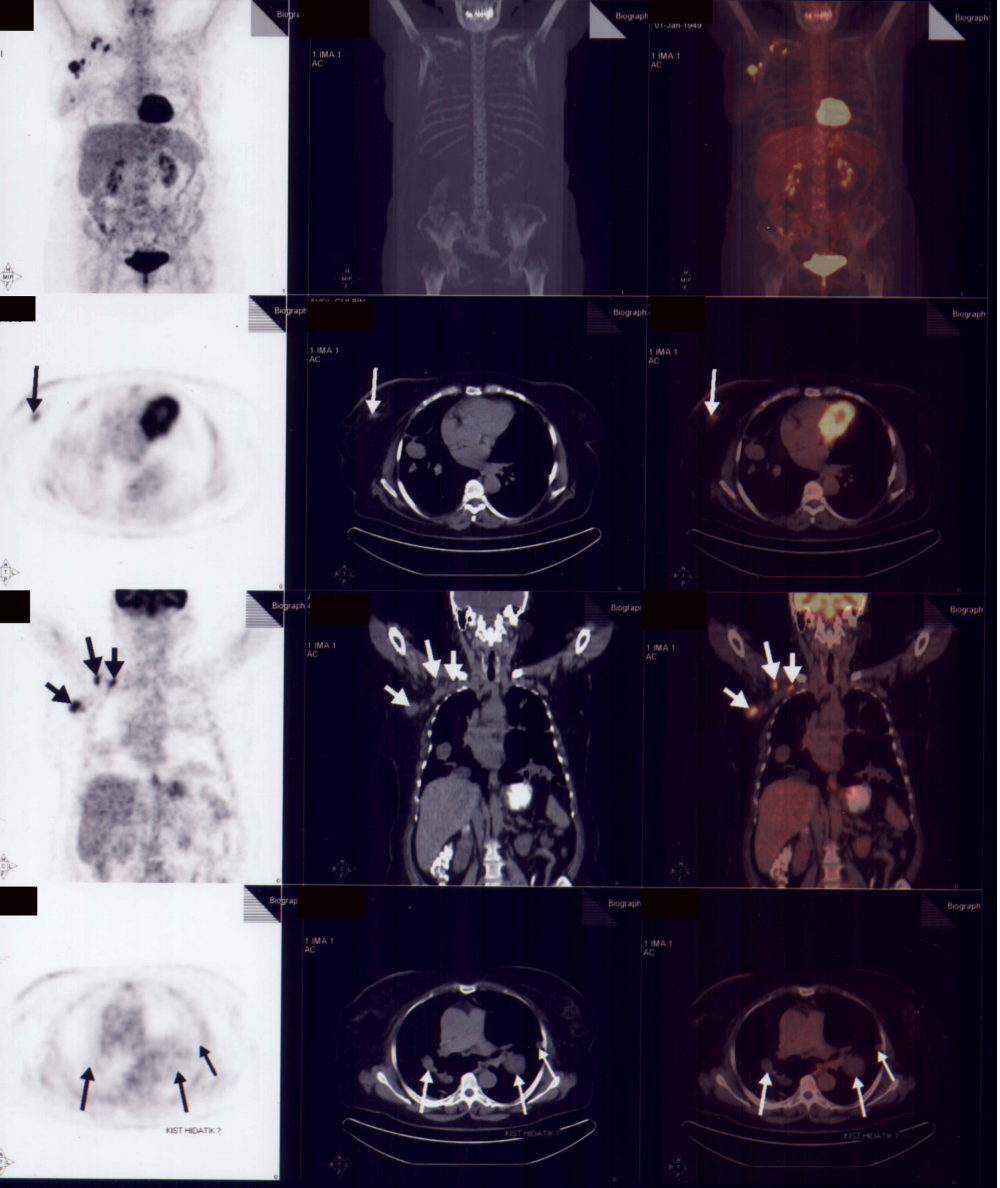
FDG PET scans, First Column of the Figure showed increased FDG uptake in both breast cancer and in metastatic axillary and supraclavicular lymph nodes (arrows) and also no FDG Uptake of the benign pulmonary lesions (small arrows).

A 56-year old woman admitted with a breast lump, chronic cough and chest pain. There was no medical or family history of note. On physical examination we discovered a palpable mass in the medial outer quadrant of the right breast and ipsilateral palpable axillary and supraclavicular lymph nodes. Mammography and ultrasound revealed a 2 cm nodule with irregular border including micro calcifications which was confirmed to be invasive ductal carcinoma by a fine needle biopsy. Chest radiography and computed tomography showed multiple nodules and masses involving both lungs highly suspicious to be metastatic in origin (Figure 1). Positron Emission Tomography (PET) scans showed increased Fluoro-deoxy-glucose (FDG) uptake in the breast cancer and axillar, supraclavicular lymph nodes as it's expected but there is no FDG uptake in pulmonary lesions suggesting a benign origin (Figure 2). She was coming from the east part of the country where hydatid disease is endemic. Positive indirect hemaglutination test supported the diagnosis of pulmonary echinococcosis. Chemotherapy for Stage IIIC breast cancer (Adriamycin, cyclophosphamid and paclitaxel) and albendazol treatment began for the patient.

## Discussion

Hydatid disease is one of the most important helminthic diseases. Echinococcosis is worldwide in distribution; it occurs most commonly in sheep and cattle-raising areas [4]. Lung is the second most common involved organ but it is hard to distinguish pulmonary echinococcosis from a variety of other pulmonary lesions especially metastatic lesions to the lung. In women, the appearance of metastasis is a frequent complication of breast cancer. The lung is the second most common organ for metastasis of breast cancer after the bone [5]. We present herein a patient with breast cancer who had also bilateral pulmonary multiple masses in different size and irregular shape which were determined by plain X-Ray and thorax CT thought to be metastasis to lung. Despite increased FDG uptake for breast cancer and metastatic lymph nodes, pulmonary nodules and masses had no FDG uptake so we excluded malignancy by FDG-PET scan and decided with a high degree of certainty that they were benign lesions. In fact there is a wide differential diagnosis for multiple nodules and masses with extensive pulmonary involvement on radiography or computed tomography, which includes pulmonary echinococcosis. But they are not as accurate as FDG PET to exclude the metastatic pulmonary lesions especially >1 cm. Needle biopsy or aspiration of the hydatid cysts since anaphylactic shock would not necessary develop when draining a pulmonary abscess or other cystic lesions rather than echinococcosis [2].

A study by Schirrmeister *et al *showed that whole body FDG PET is as accurate as panel of imaging modalities currently employed and significantly more accurate in detecting multifocal disease, lymph node involvement and distant metastasis [6]. FDG PET has been shown to have impact on the staging and management of recurrent or metastatic breast cancer in cases of suspicion and in a follow-up setting. The current oncological situation can be clarified with a single basic imaging modality [6,7].

There is no doubt that multiple pulmonary nodules may be commonly seen in patients with inflammatory diseases such as echinococcosis but we want to emphasize in this manuscript that it is hard to differentiate whether these nodules are malign or not especially in patients with malignancy. At this point FDG Pet scan can be helpful by showing the high metabolic activity of the nodules. Even though Stumpe *et al *[1] found that in patients with alveolar echinococcosis baseline PET scans showed multifocally increased FDG uptake in the hepatic lesions' periphery, liver lesions were FDG negative in patients with cystic echinococcosis.

## Conclusion

FDG-PET besides being a strong indicator of malignant lung nodules, is a reliable diagnostic test that can avert needle biopsy of lesions that do not show significant metabolic activity. During the evaluation of the malignancies which could metastasize to the lung, it must be kept in mind that the appearance of bilateral multiple pulmonary masses can also be the sign of a pulmonary echinococcosis especially in endemic areas. FDG-PET with its known high negative predictive value in characterizing indeterminate pulmonary nodules >1 cm is very helpful to characterize this kind of lesions

## Competing interests

The author(s) declare that they have no competing interests.

## Authors' contributions

YK drafted the manuscript, ÝS helped in preparing the manuscript, AÝF did literature search and helped with manuscript, MU helped with the nuclear medicine component of manuscript, MLA helped with preperation of manuscript.

All authors read and approved the final manuscript.

## References

[B1] Stumpe KD, Renner-Schneiter EC, Kuenzle AK, Grimm F, Kadry Z, Clavien PA, Deplazes P, von Schulthess GK, Muellhaupt B, Ammann RW, Renner EL (2007). F-18-fluorodeoxyglucose (FDG) positron-emission tomography of Echinococcus multilocularis liver lesions: prospective evaluation of its value for diagnosis and follow-up during benzimidazole therapy. Infection.

[B2] Morar R, Feldman C (2003). Pulmonary echinococcosis. Eur Respir J.

[B3] Erdogan A, Ayten A, Demircan A (2005). Methods of surgical therapy in pulmonary hydatid disease: is capitonnage advantageous?. ANZ J Surg.

[B4] Gencer M, Ceylan E Pulmonary echinococcosis with multiple nodules mimicking metastatic lung tumor in chest radiography. Respiration.

[B5] Bland KI, Vezeridis MP, Copeland EM, Schwartz SI (1999). Breast. Principles of Surgery.

[B6] Schirrmeister H, Kuhn T, Guhlmann A, Santjohanser C, Horster T, Nussle K, Koretz K, Glatting G, Rieber A, Kreienberg R, Buck AC, Reske SN (2001). Fluorine-18 2-deoxy-2-fluoro-D-glucose PET in the preoperative staging of breast cancer: comparison with the standard staging procedures. Eur J Nucl Med.

[B7] Siggelkow W, Zimny M, Faridi A, Petzold K, Buell U, Rath W (2003). The value of positron emission tomography in the follow-up for breast cancer. Anticancer Res.

